# When COVID-19 strikes mental health: a measurement analysis of reassurance seeking behavior scale in Peruvian population

**DOI:** 10.3389/fpsyg.2023.1132804

**Published:** 2023-04-17

**Authors:** Denisse Manrique-Millones, Jackeline García-Serna, Ronald Castillo-Blanco, Nataly Fernández-Ríos, Danny Alonso Lizarzaburu-Aguinaga, Geraldina Rebeca Parihuamán-Quinde, David Villarreal-Zegarra

**Affiliations:** ^1^Carrera de Psicología, Universidad Científica del Sur, Lima, Peru; ^2^Instituto Peruano de Orientación Psicológica, Lima, Peru; ^3^Universidad del Pacífico, Lima, Peru; ^4^Facultad de Medicina, Universidad Nacional de San Agustín Arequipa, Arequipa, Peru; ^5^Facultad de Ingeniería y Arquitectura, Universidad César Vallejo, Lima, Peru; ^6^Universidad San Pedro, Huacho, Peru; ^7^South American Center for Education and Research in Public Health, Universidad Privada Norbert Wiener, Lima, Peru

**Keywords:** measurement invariance, Peru, COVID-19, anxiety, depression, reassurance seeking behavior

## Abstract

**Background:**

The long-lasting impact of the COVID-19 pandemic cannot be overstated. To combat its dire consequences, some screening measures have been hastily developed and require robust verification to explore their adequacy across different groups. The present research study aimed to analyze measurement invariance by sociodemographic characteristics of the Coronavirus Reassurance Seeking Behavior Scale (CRSB) in Peruvian adults.

**Methods:**

A total of 661 participants completed The Coronavirus Reassurance Seeking Behavior Scale (CRSB), the Coronavirus Anxiety Scale (CAS), and sociodemographic information a subgroup filled in the Patient Health Questionnaire (PHQ-9). Reliability and measurement invariance across sociodemographic characteristics were analyzed. Likewise, associations with depression and dysfunctional coronavirus anxiety were examined.

**Results:**

Results showed that the single factor structure of the CRSB with correlated errors fitted the data adequately and the instrument was invariant across gender, age, and loss of a significant relative to COVID-19. In addition, significant associations with depressive symptoms and dysfunctional anxiety were found.

**Conclusion:**

The findings of the present study suggest that the Coronavirus Reassurance Seeking Behaviors Scale is invariant across different sociodemographic characteristics.

## 1. Introduction

Since the beginning of the coronavirus disease (COVID-19) outbreak, more than 3.5 million lives have been claimed, and the number of those infected has surpassed the 168 million mark worldwide (Center for Systems Science and Engineering, 2020), reaching almost every country in the globe.

Latin America is one of the regions hit the hardest by the COVID-19 pandemic, according to estimates by international organizations such as the Economic Commission for Latin America (ECLA), the World Bank and the World Health Organization (WHO). It has resulted in the worst economic, social and productive crisis in the region of the last 120 years, in which unemployment, poverty, and inequality have increased (Economic Commission for Latin America [ECLA], 2021).

Although Latin America had sufficient time to prepare and face the pandemic, its structural conditions in the economy, housing, and health ended up making the continent a propitious place for the spread of COVID-19 (Peñafiel-Chang et al., 2020). A health system that was not prepared for such crisis, with high hospital occupancy that in some cases has reached the limit of emergency (Agencia EFE, 2020), and a difficult economic situation in the most unequal region in the world (Martí i Puig and Alcántara Sáez, 2020), are part of an explosive cocktail.

In this context, Peru made world news. In August 2020, the southern country reached its maximum peak of deaths per day from COVID-19, becoming the one with the highest mortality rate in the entire planet, according to the ranking prepared by the Coronavirus Resource Center of the Johns Hopkins University (CNN Español, 2020). With the arrival of 2021, and despite the devastating second wave in Europe, it still continues among the top ten places on that fatality podium and second in Latin America after Mexico (Alayo Orbegozo, 2021).

Among the restraint measures that some governments have adopted in order to stop or prevent the spread of the virus are: social distancing, isolation, quarantine, lockdown (Alfano and Ercolano, 2020; Lau et al., 2020; Sánchez-Villena and de La Fuente-Figuerola, 2020), banning of social, productive and economic activities, as well as the closure of borders. Some countries have even implemented a strict nationwide curfew. These measures have a detrimental impact on mental health (Yao et al., 2020; Msherghi et al., 2021; López Steinmetz et al., 2022), increasing levels of stress, depression symptomatology and anxiety (Elmer et al., 2020; Galea et al., 2020; Lima et al., 2020; Zhang and Ma, 2020; Mendoza-Ruvalcaba et al., 2022).

### 1.1. Impact of the pandemic on mental health

Quarantine and isolation increase the occurrence of psychological and mental problems, mainly originating from the absence of interpersonal communication. Depressive and anxiety disorders are more likely to develop or aggravate (Xiao, 2020; Zandifar and Badrfam, 2020). This propensity to display difficulties ranges from isolated symptoms to the development of a mental ailment such as insomnia, anxiety, depression, and post-traumatic stress disorder (Huremović, 2019). Likewise, in the social withdrawal in which people have been forced to restrict their mobility and reduce their social contacts to a minimum, the availability of timely psychosocial interventions and routine psychotherapeutic counseling have been drastically cutdown (Xiao, 2020).

In the context of a pandemic, it is important to consider the loss of function that can accompany the acquired disease. This in turn represents demoralization and helplessness, reaching a state of mourning (Huremović, 2019). Likewise, people subjected to the stress of the outbreak may present marked anguish and a significant deterioration in social or occupational functioning, and if they persist with a sad mood, a major depressive disorder may arise. It has been postulated that the combined effect of loss and threat may explain the frequent occurrence of depression (Styra et al., 2008).

The paralyzing fear that this disease triggers could be explained by its novelty and the uncertainty it generates (Asmundson and Taylor, 2020). The substantial number of patients and suspected infected cases raised public concern about becoming infected. This situation preconditions, to some extent, people to seek reassurance by excessive searching for news and information on COVID-19.

### 1.2. Reassurance seeking behavior as a vulnerable emotional distress factor

Excessive reassurance seeking is a relevant mechanism associated with the management of psychological distress. Empirical research studies have linked it to the prolongation of anxiety including generalized anxiety disorders (Beesdo-Baum et al., 2012), obsessive-compulsive disorder (Salkovskis, 1996; Salkovskis et al., 1998) and health anxiety or hypochondriasis (Taylor and Asmundson, 2004; Halldorsson and Salkovskis, 2017). It has been conceptualized as the “repeated solicitation of safety-related information from others about a threatening object, situation or interpersonal characteristic despite having already received this information” (Parrish and Radomsky, 2010).

Reassurance seeking behavior has been appointed as a vulnerability factor that enhances emotional distress during a pandemic (Lee and Crunk, 2020). Recurrent verification and reassurance seeking can ensue as a reaction to an infection risk (Taylor and Asmundson, 2004).

People with an overly excessive concern over their health are characterized by frequent medical redundant checking and reassurance seeking (Asmundson and Taylor, 2020).

One possible explanation of why people engage in excessive reassurance seeking is their lack of tolerance toward uncertainty (Taylor, 2019). In fact, some disorders are associated to high intolerance of uncertainty (Fergus et al., 2015). Those with high levels of intolerance address their uncertainty through reassurance seeking and behaviors checking (Dugas and Robichaud, 2007). One of the ways in which they do this is by searching for medical information online or asking a professional.

Continual reassurance can also be explained under the lens of maladaptive coping (Taylor, 2019). Although the pandemic has mobilized everyone to use different ways of coping to deal with this stressful situation (Voronin et al., 2020), some people may resort to use maladaptive safety behaviors (i.e., excessive hand washing, repeatedly and unnecessarily seeking reassurance in social media or from health professionals) in an attempt to relieve their stress in the short term, but failing to resolve the underlying fears and concerns, which allows the anxiety to prevail in the long term (Wheaton et al., 2012).

### 1.3. CRSB and associated variables

In order to understand the causes of excessive distress, reassurance seeking behaviors play an important role as a vulnerable factor to emotional distress (Taylor, 2019). As a response to the pandemic, Lee et al. (2020) have developed the Coronavirus Reassurance-Seeking Behavior Scale (CRSB) which aims to measure the recurrence of engagement in coronavirus-related reassurance seeking behaviors. The CRSB is a short 5 item scale with good psychometric properties. It has good internal consistency (α = 0.90) and a solid single factorial structure (Lee et al., 2020). Although it has been translated into some other languages like Turkish, Persian, Greek and Italian, there is no formal evaluation of its equivalence across different groups.

Its original version had a high correlation with dysfunctional coronavirus anxiety, depression, among others (Lee et al., 2020). Previous research studies have mentioned that excessive reassurance-seeking is a hallmark feature in developing and perpetuating anxiety and specifically been associated to corona phobia and health anxiety (Lee et al., 2020).

Likewise, people that are prone to have a high intolerance to uncertainty and use excessive reassurance safety behaviors are likely to have high levels of depression (Thompson et al., 2010; Taylor, 2019).

### 1.4. The present study

Based on the aforementioned and in response to the pandemic situation, the aim of the present study was to evaluate whether the CRSB is equivalent across some sociodemographic variables in a sample of Peruvian adults. In addition, reliability indices were inspected, and it was hypothesized that CRSB had a positive and direct relationship with depression by means of the Patient Health Questionnaire (PHQ-9) and with dysfunctional anxiety measured by the Coronavirus Anxiety Scale (CAS).

## 2. Materials and methods

### 2.1. Participants

The sample comprised 661 Peruvian adults for which cross-sectional data was collected using a snowball sampling technique mainly in urban zones of the capital, Metropolitan Lima. Participants that did not meet the inclusion criteria were filtered out. The final sample consisted of 661 participants (59% women), their age ranged from 18 to 45 (*M*_age_ = 23.5, *SD* = 6.2). Table 1 includes the complete sociodemographic characteristics of the participants. Some variables were re-categorized for the sake of simplicity. Additionally, some categories had very few cases. In the case of age, and following some developmental theorists (Arnett, 2000, 2015; Mehta et al., 2020) two very well distinguished groups were formed, emerging adults, which ages ranged from 18 to 29 years (Arnett, 2000) and established adults, which ages ranged from 30 to 45 years (Mehta et al., 2020). Likewise, original educational level categories were collapsed to basic education (primary and secondary) and higher education (university studies onward) supported by the Peruvian educational structure and the Peruvian General Education Law (2003).

**TABLE 1:** Sociodemographic characteristics of the Peruvian sample (*n* = 661).

	*n*	%
**Gender**		
Female	392	59
Male	269	41
**Age**		
Emerging adults (18–29)	554	84
Established adults (30–45)	107	16
**Marital status**		
Single	573	86.7
Cohabiting or married	67	10.1
Divorced or separated	21	3.2
**Number of children**		
No children	573	87
Has children	88	13
**Education level**		
Basic education	407	62
Higher education	254	38
**You know people with COVID-19**		
Yes	481	73
No	180	27
**COVID-19 diagnosis**		
No	509	77
Diagnosed or convalescent	152	23
**Loss of a significant relative to COVID-19**		
Yes	237	36
No	424	64
Levels of depressive symptoms*		
None	60	34.9
Mild	31	18
Moderate	32	18.6
Moderately severe	35	20.3
Severe depression	14	8.1

*Variable depression has a sample of n = 172.

### 2.2. Measures

#### 2.2.1. Sociodemographic information

Participants were requested to state a number of background variables among the most important: gender, age, number of children, educational level, whether the participant have or have had the diagnosis of COVID-19 and whether they have lost a significant relative due to COVID-19.

#### 2.2.2. Coronavirus reassurance-seeking behavior scale

The CRBS (Lee et al., 2020) is a self-reported scale that looks to evaluate reassurance-seeking behaviors related to preoccupations over coronavirus infection. It consists of 5 items through which participants indicate how often they got engaged in reassurance-seeking behaviors (e.g., “I spoke with a medical professional about my symptoms to see if I was infected with the coronavirus disease”) in the last 2 weeks. Items are rated on a five-point Likert scale, ranging from “not at all” (0 scores) to nearly every day over the last 2 weeks (4 scores). The total score of the CRBS can range from 0 to 20. In the present study, internal consistency coefficients were adequate (α = 0.89, ω = 0.9).

#### 2.2.3. Patient health questionnaire

The PHQ-9 (Spitzer, 1999) is a brief self-administered questionnaire and consists of nine items assessing depressive symptoms (e.g., “Thought that you would be better off dead, or of hurting yourself”). Participants rated the frequency of their answers in the last 2 weeks on a 4-point Likert scale ranging from “not at all” (0 scores) to “nearly every day” (3 scores). The adapted Peruvian version of the PHQ-9 (Calderón et al., 2012) was used, and has good evidence of validity supporting one dimension factor structure (Villarreal-Zegarra et al., 2019). The total score of the PHQ-9 can range from 0 to 27. Cut-off points of 0–4 (none), 5–9 (mild), 10–14 (moderate), 15–19 (moderately severe), and 20 to more (severe depression) (Kroenke et al., 2001). In the present study, internal consistency Cronbach’s alpha coefficient was excellent (α = 0.92).

#### 2.2.4. Coronavirus anxiety scale

The CAS (Lee, 2020) is a self-rated instrument and consists of five items evaluating dysfunctional anxiety over the coronavirus. Participants indicated how frequently they experience each activity over the last 2 weeks on a five-point Likert scale, ranging from “not at all” (0) to “nearly every day over the last 2 weeks” (4). Previous empirical studies have demonstrated good psychometric properties of this brief tool in different languages: Korean (Choi et al., 2020), Turkish (Evren et al., 2020), Bangla (Ahmed et al., 2020). The validated Peruvian version of the CAS was used with satisfactory evidence of validity (Caycho-Rodríguez et al., 2020). In the present study, the internal consistency Cronbach’s alpha coefficient was adequate (α = 0.85).

### 2.3. Procedure

An institutional review board (i.e., The Research Committee of San Pedro University), provided ethical approval for conducting the research study. Participants gave their consent virtually before starting the evaluation. The evaluation was anonymous, voluntary and confidential, so the study did not represent any ethical risk to the participants. Moreover, contact information from the research team was provided in case of questions, doubts, or any additional information the participants required, during or at the end of the study.

For some instruments, the validated local versions were used and for foreign tools a translation from English to Spanish was made, followed by a back translation to assure linguistic equivalence of the instruments.

A set of self-rated questionnaires and socio-demographic information was completed by Peruvians from the general population. Participants completed the survey remotely (i.e., through an online platform) in which a link was enabled and disseminated on different social media sites. Informed consent was required. Participants were told about the anonymous nature of the research, that they could withdraw from the survey at any time without further explanation and that the information would be treated as confidential as possible for research purposes.

### 2.4. Data analysis

Evidence of internal structure validity was evaluated using confirmatory factor analysis. Maximum Likelihood with Robust standard errors (MLR) method was used, which is suitable when the number of response categories for each item is equal to or greater than five (Rigdon, 1998; Raykov, 2012). Accordingly, a set of goodness-of-fit indices were used: Comparative Fit Index (CFI) and Tucker-Lewis Index (TLI), which define adequate values as those > 0.90; the Standardized Root Mean-Square (SRMR); and the Root Mean Square Error of Approximation (RMSEA), where values <0.080 are considered adequate (Keith, 2014). Likewise, goodness-of-fit indices of the model with correlated errors were evaluated.

Additionally, a stepwise Multi-Group Confirmatory Factor Analysis (MGCFA) was used to assess nested models with progressive restrictions in the gender group. Initially, we set a base or configural model. Based on this, we added restrictions at the level of factor loadings (weak model). A non-substantial discrepancy between the two models indicates weak invariance (configural model vs. weak model). We then evaluate the strong model with restrictions at the level of factor loadings and intercepts. We then, compare both models (weak model vs. strong model). We considered a non-substantial variation in each of the previous steps described if the difference was ΔCFI < 0.010 and ΔRMSEA < 0.015 or ΔSRMR < 0.005 (Chen, 2007; Putnick and Bornstein, 2016). Associations between the CRBS and the PHQ-9 and CAS were conducted by means of Pearson’s correlation analysis. We expected a positive correlation of the CRBS with the two variables. Finally, omega (ω) internal consistency coefficients were calculated. Values greater than 0.80 were considered optimal (McDonald, 1999).

All analyses were performed using Lavaan package, Version 0.6-12 (Rosseel, 2012) in R program (Version 4.2.1). The R script is attached as Supplementary material.

## 3. Results

### 3.1. Factor structure

A confirmatory factor analysis was conducted to assess the original single factor structure in a sample of Peruvian adults. The original factor structure (model 1) yielded a poor fit. Modification indices suggested adding covariance error variance for item 2 and item 3. Therefore, model 2 was run following an adjusted model that considered these correlated errors. Optimal values for the fit indices were identified, and an adequate fit for the RMSEA index value were found (see Table 2). Theoretically, items 2 and 3 are closely related to each other and both refer to the search for information on COVID-19. Subsequent analyses were performed using this model. The standardized factor loadings of model 2 are presented in Table 3.

**TABLE 2:** Goodness-of-fit indices for the models evaluated of CRSB.

Model	χ ^2^ (*df*)	CFI	TLI	RMSEA	SRMR
Model 1	44.7 (5)	0.942	0.884	0.110	0.049
Model 2	14.8 (4)	0.984	0.960	0.064	0.025

Model 1 = Model with five-items; Model 2 = Adjusted model with covariance error between item 2 and item 3.

**TABLE 3:** Standardized factor loadings of the confirmatory factor analysis for the final model.

Item	F1
01. I took my temperature to see if I was infected with the coronavirus disease.	0.69
02. I read information on the internet to see if I had symptoms of the coronavirus disease.	0.78
03. I read or watched videos to see if I was infected with the coronavirus disease.	0.81
04. I spoke with other people about my symptoms to see if I was infected with the coronavirus disease.	0.85
05. I spoke with a medical professional about my symptoms to see if I was infected with the coronavirus disease.	0.75

### 3.2. Measurement invariance

We conducted a multi-group factor analysis, imposing progressive restrictions on structure (configural), factor loadings (weak), intercepts (strong), and residuals (strict) for all models. We found good fit indices in all cases, except in the educational level group for the configural model, which presented RMSEA somewhat above the cutoff. Measurement invariance was analyzed by gender (women and men), age (emerging adults, established adults), educational level (basic education, higher education) and loss of a significant relative (yes, no) (see Table 4). Our study found that the difference in CFI between models (configural vs weak, and weak vs strong) for all groups tested was small, ΔCFI < 0.010, and at least ΔRMSEA or ΔSRMR satisfying the cutoff for measurement invariance, with the exception of educational level. In the case of the male and female groups, the difference between strong and strict invariance was significant. On account of no straightforward interpretation for strict invariance, it was considered that there was a satisfying level of invariance to allow comparisons between males and females. So, measurement invariance was found by gender at the strong level. Similarly, results show measurement invariance by age and loss of significant relative to COVID-19 groups at the strict level.

**TABLE 4:** Measurement invariance of the Coronavirus Reassurance Seeking Behavior Scale across groups.

Group	Invariance	*X*^2^ (gl)	CFI	TLI	RMSEA	SRMR	Δ CFI	Δ RMSEA	Δ SRMR
Sex	Configural	16.4 (8)	0.988	0.971	0.057	0.023	–	–	–
	Weak	22.9 (12)	0.985	0.975	0.052	0.040	0.002	0.005	0.017
	Strong	27.4 (16)	0.984	0.980	0.047	0.041	0.001	0.005	0.001
	Strict	49.3 (21)	0.961	0.963	0.064	0.047	0.017	0.017	0.006
Age	Configural	21.5 (8)	0.982	0.955	0.072	0.024	–	–	–
	Weak	24.2 (12)	0.984	0.973	0.056	0.029	0.002	0.016	0.005
	Strong	28.1 (16)	0.984	0.980	0.048	0.029	0.001	0.008	0.000
	Strict	29.3 (21)	0.989	0.989	0.035	0.030	0.003	0.013	0.001
Educational Level	Configural	25.8 (8)	0.975	0.939	0.082	0.024	–	–	–
	Weak	28.8 (12)	0.977	0.961	0.065	0.033	0.002	0.017	0.009
	Strong	37.9 (16)	0.970	0.962	0.064	0.035	0.002	0.001	0.002
	Strict	37.3 (21)	0.977	0.978	0.049	0.036	0.004	0.015	0.001
Loss of significant relative to COVID-19	Configural	19.9 (8)	0.983	0.957	0.067	0.023	–	–	–
	Weak	27.2 (12)	0.978	0.964	0.062	0.048	0.002	0.005	0.025
	Strong	34.0 (16)	0.974	0.968	0.058	0.050	0.000	0.004	0.002
	Strict	36.9 (21)	0.977	0.978	0.048	0.049	0.001	0.010	0.001

### 3.3. Relationship with other variables

Correlation analyses was conducted with latent variables in the model. Results showed that the Coronavirus Reassurance-Seeking Behaviors latent factor correlated directly and significantly with the Coronavirus Anxiety latent factor, *r* = 0.66, *p* < 0.001. Furthermore, the CRSB correlated directly and significantly with the Depression symptoms latent factor, *r* = 0.23, *p* = 0.019.

### 3.4. Reliability

Internal consistency by means of omega coefficient were calculated for the best fitting model (model 2). Thus, coefficient presented optimal levels of internal consistency, ω = 0.86.

## 4. Discussion

We have increasing evidence that the COVID-19 pandemic eroded the mental health of millions of individuals. Different stressors associated to the outbreak such as lockdown, isolation, financial anguish, physical and social distancing, fear of contagion, concern for family and friends, and uncertainty increase the levels of maladaptive behaviors, as well as the onset of mental disorders such as anxiety, post-traumatic stress, or depression (Huremović, 2019; Taylor, 2019). The main objective of the present research study was to evaluate the measurement invariance of the Coronavirus Reassurance Seeking Behavior Scale (CRSB) across different sociodemographic variables in a sample of Peruvian adults. First, we tested the internal structure of the CRSB. Results shed light on a single factor structure of the CRSB from the original English version developed by Lee et al. (2020) in which it was necessary to add an item error covariance. Thus, two models were evaluated to better understand the factorial structure of the CRSB. The complete 5 item- scale model, and the model with correlated errors in items 2 and 3. Reliability was calculated by means of omega’s coefficient guaranteeing adequate levels of internal consistency.

In evaluating the model fit, the modification indices suggest establishing the covariance between the errors of item 2 (“I read information on the internet to see if I had coronavirus symptoms”) and item 3 (“I read or watched videos to see if was infected with the coronavirus”). The content analysis of these two items represents for the individual the emphasis on evaluating their information seeking activity on the coronavirus issue. The first item analyses the individual’s exploration on the Internet, while the second item inspects the action of reading or watching videos. In the foregoing, it is important to realize that currently the reading and watching at audiovisual material is carried out primarily through the Internet. This would result in a redundancy of these two indicators. Consequently, in this study we also present and explore the error covariance model, which in future studies or replications could motivate an adjustment in the content of any of these items.

Secondly, although previous research studies have examined the importance of the psychometric properties of the scale, it has not yet been investigated whether the scale might vary in different groups. In this sense, measurement invariance across gender was supported. This shows the first evidence of the absence of measurement bias of the CRSB, as being equally accurate for both men and women (Dimitrov, 2010). The study confirmed, through configural, weak and strong invariance, that the one-dimensional structure in both subsamples shows acceptable fit values, concluding that it provided the bare minimum necessary for a meaningful interpretation of group mean contrasts.

Moreover, results suggest the equivalence of measurement of the scale across age groups. The models (emerging adults and established adults) are equivalent in their factorial loads and intercepts. Thus, the evidence indicates that the one-dimensional model with correlated errors has attributes that make it solid and robust to differences between young and established adults, showing that the reassurance seeking behavior construct is understood in the same way across groups. Similar results were obtained regarding the group that had lost someone important through COVID-19 and those who had not. In general, our results lead to establish that population-based norms are applicable to various subgroups (i.e., gender, age, loss of significant other, etc.).

Third, regarding associations with other variables, reassurance seeking behavior was positively related to anxiety. According to previous research studies (Taylor, 2019), many people are susceptible to develop anxiety and responses such as compulsive checking and reassurance-seeking regarding potential threats.

Excessive reassurance seeking behavior has been characterized as a mechanism that plays a core role in managing psychological distress. It has been associated to anxiety and perceived general threats. In this scenario, reassurance seeking behaviors sought to immediately reduce anxiety and avoid hazardous perceived situations, episodes, or stimuli. However, it is paradoxically followed by a compulsive checking seeking response over time, perpetuating anxiety (Abramowitz et al., 2002). The study of Lee et al. (2020) revealed that reassurance seeking was highly associated to anxiety related to the COVID-19 pandemic. People that have an excessive triggered response of fear of becoming infected with the virus are prone to look for reassurance that they are not afflicted.

People with anxiety indulge in reassurance seeking behavior, hoping to minimize their feelings of uncertainty. Reassurance seeking behavior is recognized as a form of intolerance toward uncertainty, leading to higher levels of worry. This behavior is associated with pathological anxiety and has contributed to the field of generalized anxiety disorders (Dugas et al., 2001).

Concerning the depression variable, it was significantly related to reassurance seeking behavior. Although reassurance-seeking alleviates worry and uncertainty in the short term, it also prolongs depression in the long term (Joiner et al., 1999). It has been reported that if highly reassurance-seeking people perceive a negative valuation of themselves, they will begin to show depressive symptomatology.

Reassurance seeking behavior has been coined as a vulnerable factor for psychopathology, with anxiety and depression as its most common manifestations (Taylor, 2019).

Contradictory to the previous literature and foregoing research studies, Lee and Cruck (Lee and Crunk, 2020) could not find significant results of reassurance seeking as a predictor of depression. It is more likely that the PHQ-4 used with only two items measuring depression may not be sensitive enough to find significant results. However, in the present study, we can confirm a significant relationship between reassurance seeking behavior and depression, albeit with a small effect. It is important to address this issue because the prevalence of depression has increased sevenfold since the COVID-19 outbreak (Bueno-Notivol et al., 2021; Villarreal-Zegarra et al., 2023).

National, as well as international Public Health institutions are advised to address the state of general public mental health, in order to improve the wellbeing of citizens.

## 5. Limitations and conclusion

Although these findings are promising, there are some limitations worth mentioning. First, the sampling method was chosen by convenience in an effort to deal with time constraints and limited resources. In this sense, as the sample selection is not random, it is not possible to reach generalizations of the results. Future studies should use a probabilistic sample involving different regions of the country to have more accurate and categorical conclusions. Second, the study was based on self-report measures which might have some bias associated to social desirability or memory- related effects. It is recommended that studies also use other methodological strategies such as a qualitative approach (i.e., in depth interviews). Third, for the depression variable, we obtained a smaller sample size since not all participants chose to respond to this scale, possibly because it was located at the end of the survey and the answer option was left free due to the length of the entire survey. Although results yielded a significant relationship with reassurance seeking behavior, a small effect size was found.

Regardless of the shortcomings of the present study, the Coronavirus Reassurance Seeking Behaviors Scale has good psychometric properties. It can be used as a potential screening tool to identify people vulnerable to experience anxiety related to the novel coronavirus disease.

## Data availability statement

The raw data supporting the conclusions of this article will be made available by the authors, without undue reservation.

## Ethics statement

The studies involving human participants were reviewed and approved by the Research Committee of San Pedro University. The participants provided their written informed consent to participate in this study.

## Author contributions

DM-M conceptualized the study, did the project administration, and study design. DM-M, DV-Z, RC-B, and JG-S prepared the methodology and formal analysis. DM-M, NF-R, DL-A, and GP-Q collected the data. DV-Z and JG-S did data curation. DM-M wrote the first draft of the manuscript. All authors contributed to the article and approved the submitted version.
