# Supplementary material for: When COVID-19 strikes mental health: a measurement analysis of reassurance seeking behavior scale in Peruvian population
Source: Front Psychol. 2023 Apr 17;14:1132804. doi: 10.3389/fpsyg.2023.1132804 (PMC10151000; doi:10.3389/fpsyg.2023.1132804)
Supplement: Supplementary file 1 [file Data_Sheet_1.docx]

**R Script**

library("foreign")

library("psych")

library("lavaan")

library("semTools")

library("semPlot")

rm(list = ls())

Mydata=read.spss("Data_CRSB_study.sav",to.data.frame=T,use.value.labels=F)

Mydata=subset(Mydata,DeEstudio==1)

###...Models

Mymodel<-'F1 =~ CRBS1 + CRBS2 + CRBS3 + CRBS4 + CRBS5'

Mymodel<-'F1 =~ CRBS1 + CRBS2 + CRBS3 + CRBS4 + CRBS5

CRBS2 ~~ CRBS3'

#Mymodel<-'F1 =~ CRBS1 + CRBS2 + CRBS3 + CRBS4 '

#sem.fit = sem(Mymodel,ordered=names(Mydata),estimator="WLSMV",data=Mydata)

sem.fit = sem(Mymodel,estimator="MLR",data=Mydata)

summary(sem.fit,fit.measures=T,standardized=T)

semPaths(sem.fit,whatLabels="std",layout="tree",edge.label.cex=0.9,rotation=2,nCharNodes=15,

sizeLat=7,sizeMan=7,style="lisrel")

head(modificationindices(sem.fit)[order((modificationindices(sem.fit))$mi,decreasing=TRUE),],15)

#############...MI Gender ......###########

MydataG1=subset(Mydata,DeEstudio==1&Sexo==1)

sem.fit = sem(Mymodel,estimator="MLR",data=MydataG1)

summary (sem.fit, fit.measures=T, standardized=T)

MydataG2=subset(Mydata,DeEstudio==1&Sexo==2)

sem.fit = sem(Mymodel,estimator="MLR",data=MydataG2)

summary (sem.fit, fit.measures=T, standardized=T)

sem.fit.ConfigN<-cfa(Mymodel,data=Mydata,estimator="MLR",group="Sexo")

sem.fit.Weak..N<-cfa(Mymodel,data=Mydata,estimator="MLR",group="Sexo",group.equal=c("loadings"))

sem.fit.StrongN<-cfa(Mymodel,data=Mydata,estimator="MLR",group="Sexo",group.equal=c("loadings","intercepts"))

sem.fit.StrictN<-cfa(Mymodel,data=Mydata,estimator="MLR",group="Sexo",group.equal=c("loadings","intercepts","residuals"))

summary (sem.fit.ConfigN, fit.measures=T, standardized=T)

summary (sem.fit.Weak..N, fit.measures=T, standardized=T)

summary (sem.fit.StrongN, fit.measures=T, standardized=T)

summary (sem.fit.StrictN, fit.measures=T, standardized=T)

#############...MI Age ......###########

MydataG1=subset(Mydata,DeEstudio==1&Edad_grupo==1)

sem.fit = sem(Mymodel,estimator="MLR",data=MydataG1)

summary (sem.fit, fit.measures=T, standardized=T)

MydataG2=subset(Mydata,DeEstudio==1&Edad_grupo==2)

sem.fit = sem(Mymodel,estimator="MLR",data=MydataG2)

summary (sem.fit, fit.measures=T, standardized=T)

sem.fit.Config<-cfa(Mymodel,data=Mydata,estimator="MLR",group="Edad_grupo")

sem.fit.Weak..<-cfa(Mymodel,data=Mydata,estimator="MLR",group="Edad_grupo",group.equal=c("loadings"))

sem.fit.Strong<-cfa(Mymodel,data=Mydata,estimator="MLR",group="Edad_grupo",group.equal=c("loadings","intercepts"))

sem.fit.Strict<-cfa(Mymodel,data=Mydata,estimator="MLR",group="Edad_grupo",group.equal=c("loadings","intercepts","residuals"))

summary (sem.fit.Config, fit.measures=T, standardized=T)

summary (sem.fit.Weak.., fit.measures=T, standardized=T)

summary (sem.fit.Strong, fit.measures=T, standardized=T)

summary (sem.fit.Strict, fit.measures=T, standardized=T)

#############...MI Education level ......###########

MydataG1=subset(Mydata,DeEstudio==1&Nivel_Educativo_grupo==1)

sem.fit = sem(Mymodel,estimator="MLR",data=MydataG1)

summary (sem.fit, fit.measures=T, standardized=T)

MydataG2=subset(Mydata,DeEstudio==1&Nivel_Educativo_grupo==2)

sem.fit = sem(Mymodel,estimator="MLR",data=MydataG2)

summary (sem.fit, fit.measures=T, standardized=T)

sem.fit.ConfigN<-cfa(Mymodel,data=Mydata,estimator="MLR",group="Nivel_Educativo_grupo")

sem.fit.Weak..N<-cfa(Mymodel,data=Mydata,estimator="MLR",group="Nivel_Educativo_grupo",group.equal=c("loadings"))

sem.fit.StrongN<-cfa(Mymodel,data=Mydata,estimator="MLR",group="Nivel_Educativo_grupo",group.equal=c("loadings","intercepts"))

sem.fit.StrictN<-cfa(Mymodel,data=Mydata,estimator="MLR",group="Nivel_Educativo_grupo",group.equal=c("loadings","intercepts","residuals"))

summary (sem.fit.ConfigN, fit.measures=T, standardized=T)

summary (sem.fit.Weak..N, fit.measures=T, standardized=T)

summary (sem.fit.StrongN, fit.measures=T, standardized=T)

summary (sem.fit.StrictN, fit.measures=T, standardized=T)

#############...MI Loss of significant relative to COVID-19 ......###########

MydataG1=subset(Mydata,DeEstudio==1&Perdida_seresquerido_grupo==1)

sem.fit = sem(Mymodel,estimator="MLR",data=MydataG1)

summary (sem.fit, fit.measures=T, standardized=T)

MydataG2=subset(Mydata,DeEstudio==1&Perdida_seresquerido_grupo==2)

sem.fit = sem(Mymodel,estimator="MLR",data=MydataG2)

summary (sem.fit, fit.measures=T, standardized=T)

sem.fit.ConfigN<-cfa(Mymodel,data=Mydata,estimator="MLR",group="Perdida_seresquerido_grupo")

sem.fit.Weak..N<-cfa(Mymodel,data=Mydata,estimator="MLR",group="Perdida_seresquerido_grupo",group.equal=c("loadings"))

sem.fit.StrongN<-cfa(Mymodel,data=Mydata,estimator="MLR",group="Perdida_seresquerido_grupo",group.equal=c("loadings","intercepts"))

sem.fit.StrictN<-cfa(Mymodel,data=Mydata,estimator="MLR",group="Perdida_seresquerido_grupo",group.equal=c("loadings","intercepts","residuals"))

summary (sem.fit.ConfigN, fit.measures=T, standardized=T)

summary (sem.fit.Weak..N, fit.measures=T, standardized=T)

summary (sem.fit.StrongN, fit.measures=T, standardized=T)

summary (sem.fit.StrictN, fit.measures=T, standardized=T)
